# Report from the BIT’s 4th Annual World Congress of High-Tech Acupuncture and Integrative Medicine Held in Xi’an, China, 25–27 September 2017

**DOI:** 10.3390/medicines4040076

**Published:** 2017-10-19

**Authors:** Gerhard Litscher, Xiaodan Mei

**Affiliations:** 1 Head of the TCM (Traditional Chinese Medicine) Research Center Graz, of the Research Unit of Biomedical Engineering in Anesthesia and Intensive Care Medicine, and of the Research Unit for Complementary and Integrative Laser Medicine, Medical University of Graz, 8036 Graz, Austria; 2 Executive Chair of MCM-2017 and HTA & IM-2017, President, BIT Congress Inc., Dalian 116025, China

## 1. Preface

High-tech acupuncture is an example of a very successful cross-over between tradition and innovation. “The Annual World Congress of High-Tech Acupuncture and Integrative Medicine” was successfully organized on four occasions, and attracted more than 800 participants from 55 countries. It serves as an opportunity to present and discuss the state of the art of multidisciplinary approaches to the modernization of integrative medicine, especially Traditional Chinese Medicine and acupuncture.

**Figure 1:**
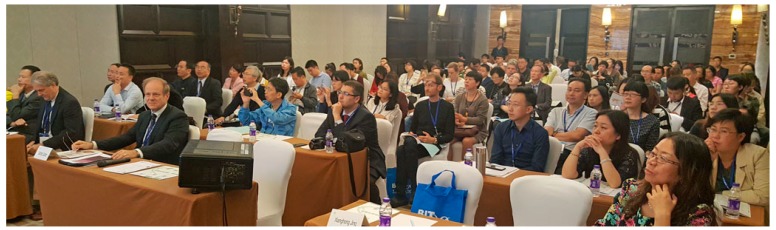
4th Annual World Congress of High-Tech Acupuncture and Integrative Medicine—2017 in Xi’an, China.

**Figure 2:**
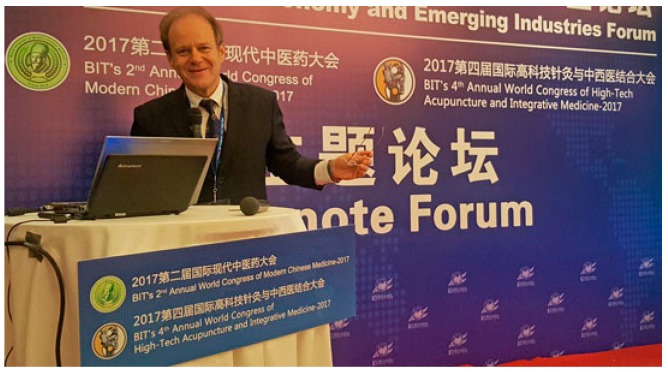
Professor Gerhard Litscher, initiator and chairman of the four (2014–2017) World Congresses of High-Tech Acupuncture and Integrative Medicine in China.

**Figure 3:**
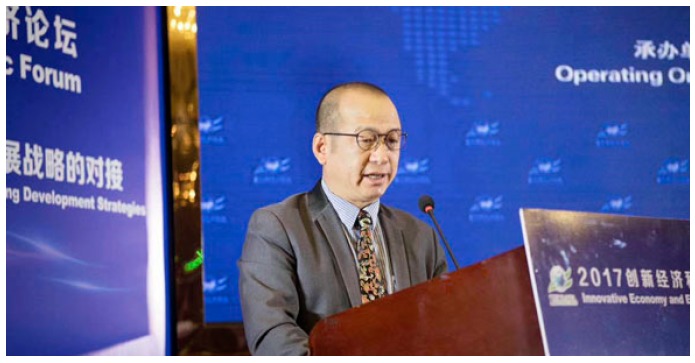
Dr. Xiaodan Mei, Executive Chair, President BIT Congress Inc.

The 4th Annual World Congress of High-Tech Acupuncture and Integrative Medicine took place in Xi’an, China on 25–27 September 2017 (Figure 1, Figure 2 and Figure 3). Themed “Exploring Different Kinds of Modern Acupuncture and Integrative Medicine,” this conference addressed the following topics:

- Modernization of Acupuncture
- Laser Acupuncture
- Integrative Laser Medicine
- Evidence-Based Complementary Medicine
- Basic Research
- Traditional and Modern Needle Acupuncture
- Electroacupuncture
- Animal Experimental Studies in Acupuncture
- Integrative Medicine

Honored leaders, renowned experts, and distinguished guests from all over the world attended the congress. Highlights of the conference were the opening ceremony, the keynote forum, the different sessions, workshops, and video demonstrations. Discussions, exhibitions, a fascinating welcome banquet, and tours also allowed for a fruitful exchange of information and ideas.

This conference is established as an annual meeting point for researchers and practitioners.

We look forward to meeting many readers of *Medicines* in 2018 at the next influential event.

## 2. Keynote Lectures

### 2.1. Acupoint Sensitization and the General Effects of Acupoints

Jing, X.

#### **Abstract** 

The structure and function of acupoints have been given more attention, especially the specific effects in treating certain kinds of diseases. Recent clinical studies only confirmed whether the effect of the selected acupoints treating the disease was better than the placebo. However, they did not show any specific correlation between acupoints and the related disease or target organs. However, the specific function of acupoints is considered the kernel of Chinese acupuncture theory. This speech focuses on the distribution of the sensitized acupoints induced by gastric mucosa injury and the local biochemistry changes of mast cells, nociceptive neuropeptides calcitonin gene-related peptide (CGRP) and substance P (SP), and allergic substances such as histamine (HA) and serotonin (5-HT). The specific effect of acupoints was related to the target organ and the sensitized acupoint reflects the function of the target organ. The acupoints also have a general effect besides the specific effect. The general effects are induced by the brain–skin axis, which is distributed in the skin and demonstrated by the activation of local HPA, neuropeptides, and cytokines.

#### **Biography** 

Dr. Xianghong Jing is the president of and a professor at the Institute of Acupuncture and Moxibustion, China Academy of Chinese Medical Sciences. She has devoted herself to understanding the effects of acupuncture and moxibustion. She has taken charge of eight research projects including the 973 Program of the China Ministry of Science and Technology, and other key projects of the National Natural Science Foundation. She has studied comprehensively and received remarkable results in acupoint general effects, acupoint sensitization, characteristics of acupoints’ dynamic process, the correlation between acupoints and viscera, and the relationship between the primary vascular system and meridians. She has published 70 papers, including 40 Science Citation Index (SCI)-cited articles.

### 2.2. Modern Ear Acupuncture—Highlights from High-Tech Research

Litscher, G.

#### **Abstract** 

Ear acupoint research and treatment has been advancing step by step worldwide. In this keynote lecture new developments and results from innovative research on auricular medicine will be presented. The introduction of lasers adds to existing stimulation with needles, electricity, pressure, and liquids an additional technique of auricular acupuncture. The latest scientific findings on ear acupuncture with lasers (infrared, red, blue, green, and yellow) will be discussed in the context of clinical applications. The auriculocardiac reflex (or vascular autonomic signal) is an important method in auricular medicine. New methodological approaches for the detection and quantification of the auriculocardiac reflex from the Medical University of Graz will be demonstrated. Even small, pulse-dependent alterations of the skin surface can be visualized. Sino-European transcontinental basic and clinical high-tech acupuncture studies demonstrate the modernization of auricular acupuncture and the scientific route from auricular therapy to auricular medicine.

The studies are supported by the Austrian Ministry of Science, Research and Economy (BMWFW) and the German Academy of Acupuncture (DAA).

#### **Biography** 

Gerhard Litscher is head of the Research Unit for Complementary and Integrative Laser Medicine, the Research Unit for Biomedical Engineering in Anesthesia and Intensive Care Medicine and the TCM Research Center of the Medical University of Graz. He is a doctor of technical and medical sciences and has published about 600 scientific papers (>200 SCI/PubMed-listed). He is the author and/or editor of 13 books and editor-in-chief and/or member of the editorial board of more than 35 international journals (e.g., association editor for Europe for *Medical Acupuncture*, *Journal of Acupuncture Meridian Studies* and *BMC Complementary and Alternative Medicine*, an editor as well as repeat guest editor and lead guest editor of *Evidence-Based Complementary Alternative Medicine*, and editor-in-chief of *Medicines and Integrative Medicine International*). He is a member of the World Health Organization’s (WHO) expert board for ‘Practice in Acupuncture’ and a current guest/honorary professor at 11 top universities, academies, and institutions in China. Address: Dr. Gerhard Litscher, Medical University of Graz, TCM Research Center, Auenbruggerplatz 29, 8036 Graz, Austria. E-mail: gerhard.litscher@medunigraz.at; http://litscher.info.

### 2.3. Recent Advances in Research on Acupuncture Mechanisms of Action Confirm Some Fundamental Principles of Traditional Chinese Acupuncture

Burgoon, T.S.

#### **Abstract** 

While modern ‘high-tech’ approaches to stimulating acupuncture points are helping clinical acupuncture practice to progress, ‘high-tech’ approaches to investigating needle acupuncture’s effects and mechanisms are also contributing in profound ways to our understanding of acupuncture. At the same time these technologically sophisticated investigations have proved some fundamental principles of traditional Chinese acupuncture practice.

The focus of this talk is three bodies of recent research on the effects of acupuncture on inflammation, gastrointestinal, and cardiovascular research. These detailed and comprehensive research efforts have utilized a complement of sophisticated techniques including immunohistochemistry, modern techniques of stimulation, and recording of somatic, central nervous system, and autonomic pathways, along with an array of modern surgical and neurosurgical techniques.

These studies confirm some of the most important and profound principles of traditional acupuncture theory and practice and provide an important foundation of communication with our non-acupuncture medical colleagues about the practice and benefits of acupuncture.

#### **Biography** 

Thomas Burgoon is the immediate past president of the American Academy of Medical Acupuncture (AAMA), the largest organization of physicians practicing acupuncture in the United States. He graduated from Vanderbilt University’s medical school in 1985 and competed residency training in internal medicine. He began studying acupuncture theory and practice in 1992 and studied with Anita Cignolini of Milan, Italy for 11 years and four years with the New York College of Traditional Chinese Medicine (NYCTCM). He is a member of the editorial board of *Medical Acupuncture,* the official journal of the AAMA, and is the chairperson of the Institutional Review Board of the NYCTCM. He is interested in Traditional Chinese Medicine theory and practice and also in the fostering of meaningful dialogue with our colleagues in regular medicine about acupuncture’s value in modern medical practice.

### 2.4. Survival Promoting Effect of Artemisinin against Oxidative Stress and Its Underlying Mechanisms

Zheng, W.

#### **Abstract** 

Artemisinin is an anti-malarial drug isolated from *Artemisia annua*. It is a Chinese medicine also known as *qinghaosu*. Artemisinin has been used in clinic for several decades in the treatment of malaria. It is safe, potent, and effective in humans. In this study, we found that artemisinin promoted the survival of various cell types such as PC12 cells, retinal neuronal cells RGC-5, and primary cortical cultured neurons from oxidative stress when we searched for the neuroprotectant from a library of active components of Chinese medicine by serendipity. Pretreatment of PC12 cells with artemisinin significantly suppressed SNP/H_2_O_2_/Aβ-induced cell death by decreasing the production of intracellular reactive oxygen species (ROS), preventing the decline of mitochondrial membrane potential, restoring abnormal changes in nuclear morphology, and reducing LDH release and caspase 3/7 activity. Artemisinin stimulated the activation/phosphorylation of extracellularly regulated protein kinases (ERK) kinase, CREB, and AMPK but it had no effect on the phosphorylation of Akt. In addition, ERK and AMPK signaling pathway inhibitors attenuated the protective effects of artemisinin, whereas the PI3K inhibitor LY294002 had no effect. Moreover, intravitreous injection of artimisinin concentration-dependently reversed the light-exposed damage of rat retinal physiological function detected by flash electroretinogram. Interestingly, artemisinin also promoted the survival of non-neuronal cells RPE from oxidative stress. Taken together, these results suggested that artemisinin is a potential neuroprotectant that is able to suppress the neuronal cell death induced by various oxidative stresses via activation of ERK/AMPK signaling. Our results offer support for the potential therapeutic application of artemisinin to prevent neuronal degenerative disorders. Supported by NFSC (31771128 and 31371088), FDCT (021/2015/A1 and 016/2016/A1), SARG, and MYRG2016-00052 from the University of Macau.

#### **Biography** 

Professor Wenhua Zheng, a principal investigator, postdoc, and PhD supervisor at the University of Macau, leads a group of scientists working on the neuroprotective effect of Artemisinin, aging and neuronal degenerative disorders, molecular mechanisms of neuronal cell survival, and new drug developments. He is also the editor-in-chief of the *Journal of Clinical and Experimental Pharmacology* and a lead guest editor or editor for several journals. A reviewer for more than 20 journals and the NSFC grant, he has also been a reviewer for the CIHR Research Grant in Canada, and an adjunct professor at RMIT University in Australia. Professor Zheng has written over 100 papers, which have together been cited over 3500 times. He is often a keynote speaker, invited speaker, or session chair at international conferences. He has also been invited to write reviews for journals like *Brain Research* (review) and *Neuropharmacology*, invited to write chapters for publishers like Nova, and invited to be the lead guest editor of journals like *Neuroplasticity*. In addition, he was invited to write the Forkhead box protein O section in a major reference book, *The Springer Reference: Encyclopedia of Signaling Molecules*, 2nd edition, 2017.

## 3. Lectures

### 3.1. Laser Acupuncture and Probiotics in School-Age Children with Asthma: A Randomized, Placebo-Controlled Pilot Study of Therapy Guided by the Principles of Traditional Chinese Medicine

Stockert, K.

#### **Abstract** 

Traditional Chinese Medicine postulates an interaction between the lung as a Yin-organ and the large intestine as a Yang-organ. The aim of this pilot study was to investigate in asthmatic school-age children whether treatment with laser acupuncture and probiotics according to Traditional Chinese Medicine offers a clinical benefit when used alongside standard medical treatment performed according to pediatric guidelines. Seventeen children aged 6–12 years with intermittent or mild persistent asthma were enrolled in this randomized, placebo-controlled, double-blind pilot study. Eight patients received laser acupuncture for 10 weeks and probiotic treatment in the form of oral drops (living nonpathogenic *Enterococcus faecalis*) for seven weeks. Nine patients in the control group were treated with a laser pen that did not emit laser light and were given placebo drops. Peak flow variability and FEV1 were measured and quality of life was assessed by a standardized questionnaire.

Laser acupuncture and probiotics significantly decreased the mean (SD) weekly peak flow variability as a measurement of bronchial hyperreactivity by −17.4 (14.2%) in the TCM group vs. 2.2 (22.5%) in the control group (*p* = 0.034). No significant effect was detected for FEV1, quality of life criteria, and additional medication. As an exploratory result, patients in the TCM group had fewer days of acute febrile infections when compared to the control group (1.14 (1.4) vs. 2.66 (2.5), *p* = 0.18).

In conclusion, this pilot study generates the hypothesis that the interactive treatment of lung and large intestine according to TCM by laser acupuncture and probiotics has a beneficial clinical effect on bronchial hyperreactivity in school-age children with intermittent or mild persistent asthma and might be helpful in the prevention of acute respiratory exacerbations. These results should be confirmed by further studies.

#### **Biography** 

Dr. Karin Stockert has been the president of the Austrian Acupuncture Society since 2014 and a lecturer in acupuncture and Chinese herbal medicine at the Austrian Acupuncture Society since 1989. She is an external expert in Acupuncture on the Master’s course in TCM at the Medical University of Vienna and a lecturer at the Medical University of Graz (SSM-TCM).

### 3.2. Acupuncture, Nerve, and Brain Stimulation for Mental Diseases: Empirical and Experimental Evidence

Zhang, Z.-J.

#### **Abstract** 

Over the past decade, acupuncture, nerve, and brain stimulation therapy has been increasingly introduced into the clinical practice of psychiatry. Professor Zhang, with his collaborators, has completed a series of clinical trials evaluating the effectiveness of acupuncture, nerve, and brain stimulation therapies (electroconvulsive therapy (ECT) and repetitive transcranial magnetic stimulation (rTMS)) for various depressive disorders, schizophrenia, insomnia, and obsessive–compulsive disorder. In this talk, he will suggest acupuncture as a form of nerve stimulation therapy that shares similar neural mechanisms with other nerve stimulation therapies. Then he will provide an overview of major findings obtained from acupuncture trials and the putative mechanisms of psychotropic effects of acupuncture and suggest the definition and classification of acupuncture therapies and acupuncture points. He also suggests several research directions on the use of acupuncture in the management of common mental disorders based on the idea of “turning your clinical services into research.”

#### **Biography** 

Dr. Zhang is a tenured professor and associate director for clinical affairs in the School of Chinese Medicine of the University of Hong Kong (HKU). He is also an honorary professor of psychiatry and family medicine at HKU. He is a vice-president of the Hong Kong Association for the Integration of Chinese–Western Medicine Limited (HKAIM), the deputy director of the Specialty Committee of Mental Diseases of the World Federation of Chinese Medicine Societies (WFCMS), and the deputy director of the TCM Psychology Specialty Committee of WFCMS. He received his Chinese medicine, acupuncture, and Western medicine training and earned his PhD in neuroscience in China. In 1994–2006, he lived in the United States to continue his research work in psychopharmacology and neuropsychiatry. His long-term research interest has been novel psychotropic agents and developing effective treatment strategies from acupuncture and herbal medicine for neurological and psychiatric disorders, including pain, anxiety, mood disorders, dementia, and schizophrenia. He is also interested in investigating the neural mechanisms of acupuncture using neuroimaging approaches. Professor Zhang has directed numerous clinical studies. He has authored over 120 original papers, reviews, and nine book chapters. He has an active clinical practice in Hong Kong and specializes in the use of TCM to treat various neurological and psychiatric problems.

### 3.3. Moving towards Solvent-Free Extraction with Pressurized Hot Water Extraction (PHWE) for Medicinal Plants

Ong, E.S.

#### **Abstract** 

Chemical constituents in botanicals or medicinal plants are known to have therapeutic purposes. Classes of bioactive or marker compounds present in medicinal plants and Traditional Chinese Medicine (TCM) include alkaloids, flavonoids, terpenes, saponins, and others. A botanical or multiple plants can be processed to become a food/health supplement, drug, medical device, or cosmetic. Aromatic plants and spices are commonly used for flavoring food, food supplements, and as a source of essential oils. To our knowledge, traditional extraction techniques that are commonly used for the chemical standardization of botanicals include Soxhlet extraction, sonication, heating under reflux, blending, and solid–liquid extraction. These techniques may require long extraction times, large amounts of samples and organic solvents, and may have negative effects on the environment and human health. At the same time, these characteristics mean that the sample treatment may become the error-prone part of the method. Hence, emerging extraction technologies such as pressurized hot water extraction (PHWE) will be discussed. The theory, principles, and applications of solvent-free extraction using PHWE with medicinal plants will be covered.

#### **Biography** 

Dr. Eng Shi Ong is on the academic staff of Singapore University of Technology and Design. He has a PhD in chemistry from the University of Bristol, UK. His main research interests lie in Green Chemistry for Analytical Chemistry and Metabolites Profiling.

### 3.4. Effectiveness of Auriculotherapy for Lower Urinary Tract Symptoms in Elderly Males

Suen, L.K.P.

#### **Abstract** 

Lower urinary tract symptoms (LUTS) commonly appear among elderly males. These symptoms include urinary retention, voiding difficulty, frequent feeling of urinary urgency, and nocturia, which negatively affect the daily functions and sleep quality of the sufferers. Auriculotherapy (AT) is a therapeutic approach in which specific points in the auricle are stimulated to achieve specific therapeutic purposes. This three-pronged randomized controlled study aims to determine the efficacy of AT for LUTS. Ninety-eight elderly males with moderate to severe LUTS were recruited and randomly allocated to the following groups: Arm 1 (*n* = 33): a combined approach of AT (i.e., laser auriculotherapy (LAT) followed by magneto-auriculotherapy (MAT)); Arm 2 (*n* = 33): placebo LAT followed by MAT; and Arm 3 (*n* = 32): placebo MAT followed by placebo LAT. Treatment was given three times a week for four weeks. Only one ear received treatment at a time, on six chosen auricular points. The subjects were assessed at the baseline, the end of the four-week treatment course, and during the one-month and three-month follow-up periods. The mean age of the participants with LUTS was 75.77 years old, with an average disease duration of 5.06 years. Within-group comparison showed significantly reduced post-void residual urine (*p* < 0.05) and increased quality of life (*p* < 0.001) in subjects of Arms 1 and 2 throughout the study period. The subjects in Arm 1 showed significantly improved sleep conditions compared to those in Arm 2 (*p* < 0.01) at the one-month follow up; and a significantly increased maximum urinary flow rate (Q_max_) and better quality of life than those in Arms 2 and 3 at the three-month follow up. The use of AT can promote bladder-emptying functions and enhance the quality of life for elderly patients with LUTS. The combined AT approach has a lasting effect in promoting the urinary flow rate of these participants.

#### **Biography** 

Dr. Lorna Suen is an associate professor at the School of Nursing and the director of the Squina International Centre for Infection Control at Hong Kong Polytechnic University. Her research interests include complementary health approaches, sleep studies, and infection control. She has published over 80 scientific articles over the years and has extensive experience in conducting randomized controlled trials using auriculotherapy in the clinical treatment of a number of chronic problems, including insomnia, lower back pain, osteoarthritic knee, and uncontrolled hypertension. She is also interested in investigating the predictive value of auricular diagnosis and has recently completed several projects on identifying the relationship of auricular reflective points with the coronary heart disease, diabetes mellitus, lower urinary tract syndrome, and metabolic syndrome. She is an editorial board member of a number of international refereed journals and is frequently invited to be a speaker at universities and international conferences and a consultant for numerous non-profit organizations in Hong Kong.

### 3.5. Complementary Trauma Therapy: Auriculotherapy and Myoreflextherapy

Bering, R.

#### **Abstract** 

Cognitive Behavioral Therapy (CBT), Eye Movement Desensitization and Reprocessing (EMDR), Psychodynamic Trauma Therapy (PTT) and Psychopharmacology (PhT) are established in the treatment guidelines for Posttraumatic Stress Disorder (PTSD). However, a subgroup of patients feels attracted to alternative or complementary therapy methods like acupuncture, meditation, physiotherapy, or diet for different reasons. Beside personal beliefs, non-responding or side effects lower the compliance and accelerate dropout rates in CBT, EMDR, PTT, and PhT. Moreover, PTSD involves a variation of neuro-musculo-skeletal syndromes such as tension headaches, shoulder and neck pain, backache, or abdomen trouble, categorized in ICD-10 and DSM-5 as comorbidities. Pain and other bodily sensations may express implicit memory storage of the trauma history. To address this, a crucial part of Trauma Therapy is the integration of complementary therapy methods. In our presentation, we have a three-step approach: First, we briefly demonstrate case reports of PTSD. Second, we introduce Myoreflextherapy (MT) and Auriculotherapy (AT) based on treatment protocols and in vivo demonstrations. Myoreflextherapy (MT) applies neuromuscular pressure point stimulation, which in turn provokes self-regulation of maladaptive body schemes. Auriculotherapy (AT) is based on the French school of ear acupuncture, which discovered the somatotopical organization of the ear. We demonstrate how visual intrusions, body sensations, and anxiety states can be reduced by the stimulation of trigger points via needles, pressure points, or light. Literature studies will be used to summarize the state of the art in terms of how effective MT and AT are considered to be. Third, we show how different programs of PTSD treatment may be supplemented by AT and MT. We conclude that progress in complementary alternative medicine should be integrated in the field of trauma therapy. MT and AT are promising candidates to accelerate the effects of CBT, EMDR, and PTT protocols, especially of somatoform and dissociative comorbidities.

#### **Biography** 

Dr. Robert Bering is the medical director of the Centre of Psychotraumatology at Alexianer Hospital in Krefeld and Cologne in Germany. Dr. Bering studied medicine and psychology and has degrees in both fields. Dr. Bering completed his clinical education as a specialist in psychiatry and psychotherapy at the Academic University Hospital of Cologne in Gummersbach. In 2005, he habilitated on his work in psychotraumatology. Today he is a professor at the University of Cologne in the field of rehabilitation. Anne Marie Vester is an acupuncturist and myoreflextherapist who has had her own practice in Copenhagen and Holsterbro, Denmark since 1992. She trained and collaborated with Dr. Raphael Nogier and Dr. Michel Marignan. Training and education are offered via her own institute in the fields of Traditional Chinese Medicine, Auriculotherapy, Posturology, and Psychotraumatology.

### 3.6. New Advances in Research on a Reconciliation Vessel for Emergency Treatment

Inchauspe, A.A.

#### **Abstract** 

When analyzing the properties of the reconciliation vessel, new questions arose about the origin of this wondrous circuit, as well as its configuration through the philosophical and cosmological basis of traditional Chinese medicine. Scrutinizing its location within Fu Hsi’s octogram and according to the Yellow Emperor’s coincidences in Ling Shu and Su Wen quotations, a plausible explanation arose from a detailed methodological work embodied in this paper. The author’s experience in cardiopulmonary revivals over many years allowed him to ponder this situation from various angles, including the comparison between legal, real, and energy deaths against the possibility of more widespread recovery after a terminal diagnosis.

#### **Biography** 

Academic Degree/Professional Title/Medical Title: 1986—Medical Doctor—University of La Plata; 1991—Specialist in Clinical Surgery—University of La Plata; 1991—Specialist in video laparoscopic surgery—University of Aachen—Germany; 1991—Specialist in video laparoscopic surgery—University of Tubingen—Germany; 1994—Specialist in video telesurgery—University Louis Pasteur—IRCAD—Strasbourg, France; 1996—Sub-specialization in video colorectal surgery—University Louis Pasteur—Strasbourg, France; 2005—Acupuncture specialist Argentinian Acupuncture Society; 2006—Professor—Department of Surgery—School of Medicine—National University of La Plata—Argentina. Workplace: Dr. Alejandro Korn Hospital, La Plata, Buenos Aires Argentina; Argentinean Acupuncture Society; Medical Sciences Faculty of La Plata University. Association Memberships: Editorial member—World Journal of Critical Care Medicine (2011–2019)—Beijing, China; Chairman of World Traditional Medicine Congress (2014)—Beijing, China; Invited Foreigner Professor—China National Academy of Medical Sciences (2014)—Beijing, China; Editorial member—Journal of Acute Disease (2016)—Haikou, Hainan, China; Invited Foreigner Professor—China National Academy of Medical Sciences (2016)—Beijing, China. The Main Academic Post: Member of the Investigation Department in “Dr. Alejandro Korn” Hospital, La Plata; Professor in the Argentinean Acupuncture Society; Surgery Professor, Medical Sciences Faculty of La Plata University.

### 3.7. Successful Acupuncture Treatment of Xerostomia after Chemotherapy and Radiation Therapy for Head and Neck Carcinomas

Haid, M.

#### **Abstract** 

Carcinomas of the head and neck constitute 3–5% of all the cancers diagnosed annually in the United States. Radiation therapy is applied in all but the very earliest stages of these diseases. Xerostomia occurs in 90% of these patients and is severe in 30%. This can produce crippling effects on the recipients’ quality of life, for which conventional allopathic medicine does not have any remedy. In the recent past, intravenous amifostine was administered at considerable expense and toxicity but was abandoned due to a lack of efficacy. Using a modification of the M.D. Anderson Xerostomia Index, which is referred to here as the Sheboygan Xerostomia Index, two patients with significantly symptomatic xerostomia, following combined chemotherapy and radiation therapy for squamous cell carcinoma of the head and neck, were treated with acupuncture using a combination of body and ear acupuncture: body points: LI-1′, LU-7, ST-36, CV-24; auricular points: Shen Men, Salivary Gland 2′, Point Zero. Responses were seen within one week. The first patient was treated weekly with acupuncture on weeks 1, 2, 3, 4, and 5. The second patient was treated weekly with the same points only on weeks 1, 2, 3, and 4. Their weekly Sheboygan Xerostomia Index scores declined from their pretreatment baseline (maximum possible score of 60) as follows: patient 1: 30, 23, 21, 12, 6, 7; patient 2: 34, 23, 11, 10. Their remissions were still continuing after more than a year of observation. Acupuncture can be used successfully to treat chemoradiation therapy-induced xerostomia at the community level and is cost-effective. This information should be shared with the oncology and acupuncture communities, which, by and large, are not aware of this treatment opportunity.

#### **Biography** 

Dr. Max Haid is the medical director of Sheboygan Acupuncture LLC. He is board certified in internal medicine, medical oncology, medical acupuncture, and Chinese Herbal Medicine. He is the author of more than 50 publications and was an assistant professor of clinical medicine at Northwestern University Medical School. He has particular interests in integrative oncology and indigenous healing traditions.

### 3.8. Integration of Acupuncture Research with Modern Science—From Observation to Prediction

Shang, C.

#### **Abstract** 

Based on the gold standard of science, successful integration of acupuncture research with modern science should lead to a model that has multiple independently confirmed predictions, ideally in both acupuncture research and conventional biomedical research. A recent literature review showed that the growth control organizer model of acupuncture meridian system has achieved this goal. This model suggests that acupuncture points originate from organizers in embryogenesis.

The following predictions of the model have been independently confirmed: (1) Acupuncture has extensive growth control effects. (2) Singular point and separatrix exist in morphogenesis. (3) Organizers have high electric conductance, high current density and high density of gap junctions. (4) High density of gap junctions is distributed as separatrices or boundaries at body surface after early embryogenesis. (5) Many acupuncture points are located at transition points or boundaries between different body domains or muscles, coinciding with the connective tissue planes. (6) Many morphogens and organizers continue to function after embryogenesis. (7) The effect sizes of real acupuncture over sham acupuncture are negatively correlated with the disease severity, patient’s age, and the number of acupoints used.

This is the first biological–physical model of acupuncture that can predict and guide clinical acupuncture research. It has outlined the origin, structure, and function of acupuncture points and meridian system with multiple independently confirmed predictions—meeting the gold standard of science (https://www.ncbi.nlm.nih.gov/pmc/articles/PMC2644274/; http://www.sciencedirect.com/science/article/pii/S0079610716300487).

#### **Biography** 

Dr. Shang is an adjunct professor of medicine at Baylor College of Medicine and a practicing physician at the Permanente Medical Group. He is a recipient of the Medical Acupuncture Research Foundation Special Award. He is on the editorial board of *Medical Acupuncture*, the world journal of acupuncture–moxibustion. He is on the University Cooperation Working Committee of the World Federation of Acupuncture Moxibustion Societies. He has been an invited reviewer for the *New England Journal of Medicine*, *Annals of Internal Medicine*, and *British Medical Journal*. Dr. Shang has given lectures on acupuncture research at various institutions such as Harvard Medical School, New England School of Acupuncture, Peking University, and Zhongshan University.

### 3.9. Diagnostic Importance of the Tender Points of Migraine and Tension-Type Headaches Using Gold Acupuncture and Meridian

Park, K.H., Park, S.W., and Yoo, T.W.

#### **Abstract** 

Background: It is very difficult to decide the location of a headache through history taking; this causes unsatisfactory results in diagnosis and treatment. Checking the pain site through presently used diagnostic methods is not standardized. In Korean Hand Therapy, there are Acupuncture and Micromeridian Points on the hand. Gold Acupuncture and Meridian Points were developed based on Micromeridian system. We propose using Gold Acupuncture and Meridian Points.

Methods: This procedure was performed during physical examinations at the Department of Neurology, Pusan National University Hospital from March 2009 to February 2013. Six hundred patients with a primary headache, without other neurological or systemic diseases, were included. We checked tender points using Gold Acupuncture Points. Results: There were several patterns of tension-type headache and migraines. We divided the groups into pure migraine (right, left, and both sides), pure tension type (right, left, and both sides), and mixed form. The locations of pure migraines were on the right side, left side, or both sides. The locations of pure tension type were also right side, left side, or both sides. The locations of mixed type were in various patterns. 

Conclusions: It is very important to pinpoint the location to diagnose a primary headache. It is not easy to locate the pain side/sites in practice. We might need to develop a new protocol to decide the location if we apply the New Acupuncture and Meridian System (Gold Meridian) to the head and neck. We can improve the diagnostic value of migraines, tension-type headache, and mixed-type headache, including some chronic migraines, using a new method that is easy to implement.

#### **Biography** 

Kyu Hyun Park, MD, PhD was a professor of neurology from 1989 to 2014 and is an emeritus professor at Pusan National University, Busan, Korea. He has worked at the Department of Neurology, Jungang Nara Hospital since 2016. He was a vice-president of the Korean Integrative Medicine Association. He received his MD from the School of Medicine, Pusan National University in 1974 and a doctorate in internal medicine in 1984. He got his license of internal medicine in 1982 and his license of neurology in 1984. He was a research fellow at the Clinical Electrophysiology and Muscle Laboratory UAB in the USA in 1987. He is a former president of the Korean Neurological Association and a former director of the Longevity Science of Technology and Institute (LSTI) of Pusan National University. He has studied acupuncture since 1968, and Korean Hand Acupuncture since 1982. He has membership in the National Academy of Medicine of Korea, the Korean Association of Internal Medicine, the Korean Neurological Association, the Korean Geriatric and Gerontology, the Korean Dementia Association, the International Headache Society, and the Korean Hand Acupuncture. His fields of interest are neuroelectrophysiology, cerebral blood flow, headaches, strokes, movement disorders, dementia, and acupuncture, especially Koryo Hand Therapy.

### 3.10. How Is TCM Combined with Western Medicine, and What Is Integrative Medicine?

Pan, W.

#### **Abstract** 

Concomitant with the limitations of Western medicine, there is a new type of additional medicine, namely integrative medicine (IM). Since the 1950s, in China, IM has been defined as Traditional Chinese Medicine (TCM) combined with Western medicine. The Chinese Tu Youyou received the Nobel Prize in Medicine in 2015 for her excellent integrative work in malaria research. Most international researchers think this method is the only meaning of IM. In fact IM is a big concept that contains any treatments that could improve physical and mental health in patients with various diseases and can benefit patients with many diseases. It may include traditional medicine (Traditional Chinese Medicine, traditional Indian medicine, Ayurveda, etc.), acupuncture and moxibustion, manipulation, tai chi chuan, yoga, meditation, mind therapy, vitamin therapy, and medicinal diets. Thus, the concept of IM is somewhat narrow and should be termed ‘specialized IM’ (SIM). IM now contains all aspects of health care needs including modern medicine (Western medicine). If the concept is expanded, it should be called ‘generalized IM’ (GIM) or modern IM. GIM has been recommended in a large tertiary care hospital with conventional application. It is becoming more common in U.S. health care. The real “integrative” medicine should not only mean “plus” but “inter,” “mix,” and “combine”; finally, a new method or medicine will be created. This new medicine is IM. GIM should not be confined to treating diseases only with modern medicine, TCM, psychotherapy, meditation exercises, health care products, or SIM. We should also make great efforts to utilize the huge potential of CAM, traditional medicine, and modern technology to identify effective means of diagnosis, quantitative evaluation, and precision treatment of diseases.

#### **Biography** 

Professor Weidong Pan is the associate director of the Department of Neurology at Shuguang Hospital, affiliated with Shanghai University of TCM. He received a bachelor’s degree in TCM from the College of Traditional Chinese Medicine, Ningxia Medical University, more than 20 years ago. He studied clinical neurology at Tokyo University, Tokyo, Japan and got his doctoral degree and post-doctoral research experience there. Now he is the chairman of the neuroendocrine committee of the Shanghai Integrative Medicine Association (since 2015), the vice-chairman of the Shanghai Immune Association (since 2015), the vice-chairman of the vertigo disease committee of the Shanghai Integrative Medicine Association (since 2017), a member of the Chinese Society of Neuroscience, and a member of the Society of Chinese Physicians Association. He is also the editor-in-chief of the journal *Integrative Medicine International* (IMI), the executive editor of the *Journal of Neurology and Neurological Rehabilitation* (JNNR), and an editorial member of many international journals. He has published more than 100 articles about Traditional Chinese Medicine and integrative medicine in Chinese, English, Japanese, and Russian.

### 3.11. Quality of Life of Patients Seeking Acupuncture Treatment in Brazil

Silva-Filho, R.d.C.

#### **Abstract** 

Introduction: Acupuncture has been shown to treat diseases, alleviate symptoms, and increase general wellbeing in different complaints and the evaluation of quality of life can be an important index for the practitioner as well as for the patient.

Objective: Compare the quality of life in two groups of people seeking treatment from the acupuncture clinic of the Brazilian School of Chinese Medicine, using the WHOQOL-BREF instrument from the World Health Organization.

Methods: 100 people were randomly selected and separated into two groups: aesthetic group N (50) and general outpatient group N (50). After informed consent was obtained, everyone answered the WHOQOÇ-BREF questionnaire. The data collected were statistically analyzed and the groups were compared through the non-parametric Mann–Whitney test and also through the *t*-student test for comparing the independent domains. The confidence level used in the analysis was 95%.

Results: The results showed that subjects from the aesthetic group were more satisfied with their health (Q2), walking ability (Q15), ability to perform daily activities (Q17), and ability to work (Q18) than subjects in the general outpatient group. Patients in the general outpatient group were prevented by physical pain from doing what they needed to (Q3) and reported needing more attention from a doctor to undertake everyday life activities (Q4), but were more able to accept their physical appearance than patients in the control group.

Conclusion: The WHOQOL-BREF instrument was effective at demonstrating the differences in quality of life of subjects seeking acupuncture treatment.

Keywords: acupuncture; quality of life.

#### **Biography** 

Reginaldo de Carvalho Silva Filho is the founder and president of the Brazilian College of Chinese Medicine. The Brazilian College of Chinese Medicine was founded in 2001 and is now the largest institution focused on Chinese Medicine teaching, treatment, and research in Brazil. It is located in Sao Paulo, the largest city of Brazil, with another 15 branches in different parts of Brazil aiming to spread acupuncture and Chinese Medicine theories and practice based on our motto: “Tradition and Modernity.” Other achievements: chief editor of *Brazilian Journal of Chinese Medicine*. Executive member of Presidium of World Federation of Chinese Medicine Societies (WFCMS). Associate professor of WFCMS. Guest professor at Chengdu University of Chinese Medicine. Guest Professor of Higher School of TCM (Fundación Europea de Medicina Tradicional China), Spain. Guest Lecturer of JiangXi University of Chinese Medicine. Vice-president of WFCMS Specialty Committee of External Treatment Methods and Technologies. Vice-president of WFCMS Specialty Committee of Palliative Care for Cancer. Author and translator of about 15 different books in the Chinese Medicine field. PhD candidate at Shandong University of Chinese Medicine under the tutorship of Professor Gao Shu Zhong.

### 3.12. Are Acupuncture Guidelines Adequate with Regards to the Time Needles Are Left in the Patient?

Ling, H.W.

#### **Abstract** 

Many acupuncture schools teach that we should leave the needles in the patients for a certain time; however, this practice may not be beneficial for every patient depending on their energy imbalance. Studying the literature, the needles are frequently not maintained in patients with *Qi* deficiency, and in cases where sedation is necessary, needles are maintained.

Four acupuncture guidelines were reviewed from Australia, the UK, the USA (California), and Brazil. They were concerned with the time recommended to leave needles in a patient, and with the energy imbalances in a group of randomly selected patients.

Medical records were selected from 101 patients undergoing acupuncture using a random method involving the initials of their first names. The name, sex, age, and energy imbalance (deficiency of *Yin* or *Yang*, or *Qi*, or Blood, or Heat retention, or a combination of these energies) was noted. The analysis demonstrated that Australia and the UK do not offer references to session duration or the time needles are left in. In California, they recommend between 20 min and one hour for the sessions. In Brazil, the timing is generally 15 to 20 min. A study of the patients selected showed that 100% had some kind of energy deficiency. The most common were *Yin* and *Yang* (17.82%); *Yin*, *Yang*, and Blood (12.87%); *Yang* and Blood (10.89%). In this study we demonstrated that determining the type of energy imbalance of each patient was necessary to know if the needles needed to be maintained or not. Acupuncture guidelines could possibly leave this issue up to the practitioner’s discretion, and not give a strict reference for all patients.

#### **Biography** 

Dr. Huang Wei Ling is a Taiwan-born, Brazilian doctor (immigrated to Brazil at the age of 1; graduated in Brazil in 1992) specializing in infectious diseases, enteral and parenteral nutrition, acupuncture and pain management, Chinese herbal therapy, Chinese dietary nutrition, and homeopathy. Since 2007, she has presented her research in acupuncture, Traditional Chinese Medicine, and, more recently, homeopathy linked with TCM, at conferences around the world. She has been working in her own medical acupuncture and pain management clinic in Brazil since 1997.

### 3.13. Targeted Expression of MiR-7 Operated by a TTF-1 Promoter Inhibited the Growth of Human Lung Cancer through the NDUFA4 Pathway

Xu, L.

#### **Abstract** 

Targeted expression of genes is an important therapeutic strategy for lung cancer. MicroRNA-7 has been well documented as a promising tumor suppressor, but has never been tested in specific gene promoter-targeted expression in cancer gene therapy. Here, we first evaluated the efficacy of miR-7 expression operated by the promoter of TTF-1, a lineage-specific oncogene in lung cancer, in vitro, using a eukaryotic vector of TTF-1 promoter-operated expression of miR-7 (termed p-T-miR-7). Interestingly, using a nude mice model, the growth and metastasis of human lung cancer cells in vivo were significantly reduced in remote hypodermic injection of the p-T-miR-7 group, accompanied by increased expression of miR-7 and reduced transduction of the Akt and Erk pathways in situ. Global gene expression analysis showed that downregulation of NDUFA4, a novel target of miR-7, contributed to the effects of miR-7 expression operated by the TTF-1 promoter on the growth and metastasis of human lung cancer cells, as well as altered transduction of the Akt and Erk pathways. Finally, there was no significant difference in the weight or histopathology of other organs. These data provided a basis for the development of a novel modality of miRNA-based targeted expression therapy against lung cancer.

#### **Biography** 

Professor Xu is the head of the Research Unit for Translational Medicine and a doctor of basic medicine at Zunyi Medical College. He has had several research stays and given international lectures. He is the author of about 100 scientific publications, some on basic cancer gene therapy and tumor immunology research, as well as three books, and is currently a guest editor and/or reviewer for more than 10 international scientific journals (e.g., guest editor for *Clinical and Developmental Immunology*, reviewer for *Molecular Therapy* and *Nano Research*, etc.). Professor Xu’s special interest is miRNA-based cancer gene therapy research. He is a professor at Zunyi Medical College and a visiting/guest professor at three universities in China. He is the vice-president of the Society of Immunology, responsible for Guizhou Province, China.

### 3.14. Low-Level Laser Therapy’s Human Health Impacts: Mechanism and Efficacy

Wu, J.-H.

#### **Abstract** 

Low-level laser therapy (LLLT) and laser acupuncture (LA) have been popular all over the world. The mechanisms and efficacies of LLLT or LA are very difficult to understand completely. However, after many researchers’ struggles, the evidence on phototherapy has been gradually disclosed. These studies can be classified into three fields: the molecular level, animal models, and human clinical trials. Photo-stimulation or photo bio-modulation is based on the interaction of light with biological systems. The experimental results from the area of molecular photomedicine are widely used in clinical practice, e.g., phototherapy or LLLT. Some mechanisms of phototherapy were reviewed and summarized in this presentation. From the adjustment of the autonomic nervous system with laser acupuncture to the brain wave stimulation with laser array, it seems there are different conduction paths in the human body, like nerve conduction and meridian conduction pathways. The relationships of molecular-level experiments and clinical applications will be discussed.

#### **Biography** 

Professor Jih-Huah Wu received a PhD in Optical Sciences from the National Central University in Taiwan in 2005. After serving one year as a post-doctoral fellow in the Department of Power Mechanical Engineering at National Tsing Hua University, he joined Ming Chun University as an assistant professor in the Department of Biomedical Engineering. He became an associate professor in 2010. He was promoted to a full professor in 2014. His research interests include laser acupuncture, electro-optical system design, and light therapy. Since 2005, he has been engaged in studying laser acupuncture and low-level laser therapy.

### 3.15. Effect of Acupuncture with Strengthening the Spleen, Nourishing the Kidney, and Dredging the Governor Vessel on Motor Function and ADLin Patients with Spastic Cerebral Palsy

Liu, Z.

#### **Abstract** 

Objective: To investigate the effect of acupuncture with strengthening the spleen, nourishing the kidney, and dredging the governor vessel on motor function and ADL in patients with spastic cerebral palsy.

Methods: 120 cases of spastic cerebral palsy were divided into a control group and an observation group according to the order of treatment, with 60 cases in each group. In the control group, physical therapy and hand function training were adopted. In the observation group, acupuncture with strengthening the spleen, nourishing the kidney, and dredging the governor vessel were adopted. Acupuncture was given once every two days for 20 days per session; there were 20 days between sessions and three sessions were required in total. Separately before and after treatment, the GMFM, Peabody fine motor skills, and ADL were used to evaluate clinical efficacy.

Results: Before and after the treatment of control group GMFM, Peabody fine motor, and ADL were statistically significant (*p* < 0.05); before and after the treatment, the observation group GMFM, Peabody fine motor skills, and ADL was statistically significant (*p* < 0.01); after treatment, two groups compared GMFM, Peabody fine motor skills, and ADL were statistically significant (*p* < 0.05).

Conclusions: Acupuncture with strengthening the spleen, nourishing the kidney, and dredging the governor vessel therapy can effectively improve the gross motor function, fine motor function, and ADL ability in children with spastic cerebral palsy.

Keywords: Spastic Cerebral Palsy, Gross Motor Function, Fine Motor Function, ADL.

#### **Biography** 

Professor Liu Zhen-Huan is the deputy manager of the Affiliated Maternity and Child Care Hospital of Guangzhou University of Chinese Medicine. He is the director of the Child Rehabilitation Hospital, and a doctoral tutor at the Guangzhou University of Chinese Medicine. Professor Liu became one of the pediatricians who received the special state council allowance in China in 1994, and was given the title of a prominent youth expert by the Healthy Ministry of China in 2002. In addition, Professor Liu is an adjunct professor at the Chinese University of Hong Kong and Pharmacy and the Health Institute of Indonesian Ministry of Health. Professor Liu is known as a pediatric neurology/acupuncture doctor throughout the world.

Professor Liu has more than 30 years’ experience specializing in TCM–Western pediatrics studies and 20 years’ experience in rehabilitation for cerebral palsy. He founded the Rehabilitation Centre for Cerebral Palsy of the Affiliated Maternity and Child Care Hospital of Guangzhou University of Traditional Chinese Medicine, which is a world-renowned medical department. Since its foundation, it has received more than 30,000 children with mental retardation and cerebral palsy from many countries, including China, the United States, Japan, the United Kingdom, New Zealand, Singapore, and Poland. Professor Liu is also actively involved in the academic world. Since 1993, he has been hosting seminars and academic exchange programs with world-renowned scholars from Oxford and Cambridge Universities in the field of the application and assessment of acupuncture treatment for brain and cerebral palsy rehabilitation in children in the United States, the United Kingdom, Spain, Australia, Norway, and so on.

### 3.16. The Effect of Auricular Acupressure on Nausea and Vomiting Caused by Chemotherapy among Breast Cancer Patients

Varaei, S.

#### **Abstract** 

The aim of this study was to determine the effect of auricular acupressure in relieving nausea and vomiting among women who received chemotherapy. Forty-eight women suffering from breast cancer and receiving chemotherapy were recruited for the study. The patients were randomly assigned into two groups, experiment and control. In the initial phase of chemotherapy, the experimental group received standard medication to control nausea and vomiting and auricular acupressure for five days. Meanwhile, the control group received only the standard medications. The use of auricular acupressure led to a decrease in the number and intensity of nausea and vomiting incidents in both the acute and delayed phases in the experimental group, significantly lower than the control group (*p* = 0/001). It is suggested that nurses use this pressure technique as a complementary treatment, since it is a non-pharmacological, inexpensive, and non-invasive approach to the relief of chemotherapy-induced nausea and vomiting.

#### **Biography** 

Assistant Professor Shokoh Varaei is the head of the medical surgical and basic science department at the Nursing and Midwifery School, Tehran University of Medical Sciences. She has a PhD in nursing education and has had several research stays and international lectures, as well as about six thesis and scientific publications, some on herbal therapy and aroma therapy, and is responsible for teaching complementary medicine and the nurse’s role in this era. 

### 3.17. Brain Function Analysis Based on EEG Signal under Magnetic Stimulation at Acupoints

Guo, L.

#### **Abstract** 

Acupuncture is based on the theory of Traditional Chinese Medicine. Its therapeutic effectiveness has been proved by clinical practice for thousands of years. However, its effect mechanism is still unclear. The aim of our work is to reveal the effect mechanism of acupuncture using the methods of modern science and technology. This keynote lecture focuses on the latest research findings about acupuncture and brain function, including the effects of brain evoked potentials with the frequency and intensity of magnetic stimulation; a comparative analysis of brain evoked potentials under magnetic stimulation at acupoints and non-acupoints; the source localization of brain evoked potentials under magnetic stimulation at acupoints; the complexity analysis of EEG under magnetic stimulation at acupoints; the corresponding relationship of magnetic stimulation at acupoints with brain functional areas; the construction and topology analysis of brain functional networks based on EEG under magnetic stimulation at acupoints; and analysis of brain function under magnetic stimulation at acupoints for fatigue and insomnia population.

#### **Biography** 

Professor Lei Guo is the associate dean of the Biomedical Engineering Department at Hebei University of Technology. A PhD, doctoral supervisor, senior member of the China Biomedical Engineering Society, and visiting scholar in the USA, Professor Guo has long been engaged in biomedical engineering. His fields of interests are neural engineering, medical image processing, and machine learning. He has published more than 30 research papers in academic journals indexed by SCI and EI as the first author. He has undertaken many scientific research projects for the National Natural Science Foundation of China, the Hebei Province Natural Science Foundation, the PLA General Armament Department Project, etc.

### 3.18. Motor Function Rebuilding of Paralyzed Limbs Based on the Neural/EMG Signal Communication Principle and Electro-Acupuncture 

Wang, Z.-G. and Lü, X.-Y.

#### **Abstract** 

In this talk, the concept of exploring the signal transmission along meridians is developed. The physical nature of the meridians based on electrical network theories is presented and the results and analysis of a series of experiments on meridian lines are given. It will be shown that signals of the point-in/point-out and the signals along a non-meridian path with the same distance are significantly different. Then, it is demonstrated that an electric signal can be applied to a pair of acupoints to cause finger extension or flexion. Through experiments we have found a series of acupoints for the extension and reflex movements of the five fingers. Finally, the concept of motor function rebuilding based on the principle of neural/EMG signal communication and the functional electrical stimulation (FES) or electro-acupuncture technique is introduced. A prototype of a two-channel apparatus of eletromyographic bridge (EMGB) type has been designed, tested for product registration inspection, and used in clinical experiments in 10 hospitals. With this apparatus, a paralyzed wrist or finger can be stimulated to extend or flex under the control of a healthy arm, hand, or finger, where surface electrodes were used to detect the controlling signals and both surface electrodes and needle electrodes were used for FES. Paretic limbs and fingers of more than 60 hemiplegic patients have been treated. The rating scales have quantitatively shown that the EMGB apparatus has significantly better effects in motor function rehabilitation of paralyzed limbs than the existing FES apparatus.

#### **Biography** 

Professor Dr. Zhi-Gong Wang was born in Henan, China. He received a Master’s degree in radio engineering from Nanjing Institute of Technology (now Southeast University), Nanjing, China, in 1981, and a doctorate in electronic engineering from Ruhr-University Bochum, Germany, in 1990. From 1977 to 1981, he worked on radio communication techniques and computer-aided circuit designs for the Nanjing Institute of Technology. In 1982–1984 he worked as a lecturer in semiconductor circuit techniques at Tongji University, Shanghai. From 1985 to 1990, he worked on high-speed silicon bipolar circuit designs for multigigabit/s optic fiber communication at Ruhr-University Bochum, Germany. From October 1990 to September 1997, he was with the Fraunhofer Institute of Applied Solid State Physics, Freiburg, Germany, working on high-speed GaAs ICs for optic-fiber data transmission and MMICs. Since October 1997, he has been a full professor at Southeast University, Nanjing, China. He is the author or co-author of 20+ books, 700+ SCI/EI/ISTP-indexed papers, and is the inventor of 40+ patents in China, Germany, Europe, the USA, and Japan. He has been a senior member of IEEE since 1993, and is the chairman of the Advisory Committee of Electrical and Electronical Basic Courses of Chinese Universities and a member of the Chinese Expert Committee of Internet of Things for Healthy. He is a guest professor at 20+ universities in China, Canada, and Australia. Recently, he has been involved in IC design for optic-fiber transmission systems, RF wireless, microwave, and millimeter wave applications, and in micro-electronic systems for biomedical applications. Since 2006, he has also been interested in the principles and function of meridian and acupoints, especially for motor function rebuilding of paralyzed limbs.

### 3.19. Catgut Implantation at Acupoints for Allergic Rhinitis: A Randomized, Sham-Controlled Trial

Li, X.

#### **Abstract** 

The safety of catgut implantation at acupoints to treat allergic rhinitis (ICD-10 code J30.4) remains controversial. Here, we used a sham catgut implantation group to determine whether catgut implantation at acupoints is an effective and safe treatment for allergic rhinitis. A randomized double-blind clinical trial, with parallel groups, was conducted. The results showed an improvement of the visual analogue scale (VAS) and Rhinoconjunctivitis Quality of Life Questionnaire (RQLQ) scores in both the active and sham-controlled groups after treatment in the self-control analysis. The RQLQ scores significantly differed between the two groups after four weeks of treatment (*t* = −2.045, *p* = 0.05); this difference lasted until the end of the eight-week follow-up (*t* = −2.246, *p* = 0.033). The findings suggest that catgut implantation at acupoints is an effective and safe method for symptomatic treatment of allergic rhinitis.

#### **Biography** 

Xinrong Li, PhD and assistant professor of Otolaryngology, Chengdu University of Traditional Chinese Medicine, is an expert on the Allergic Committee of the China Society of Integrated Traditional Chinese and Western Medicine, as well as the editor of the magazine *Chinese News* and *Reviews of Otolaryngology*. Dr. Li’s special interests are the mechanism and clinical study of the allergic diseases of the upper airway and self-management education and regular practitioner review for patients with allergic diseases of otorhinolaryngology.

### 3.20. Definite Pulse Pattern for Thyroidism and Acupuncture Healing Swasthya Santulan Medicare Pvt. Ltd. India

Satarkar, S.

#### **Abstract** 

Basic research on pulse diagnosis and pulse patterns for accurate diagnosis in acupuncture and integrative quantum healing approaches has been successfully performed on all chronic illnesses. Hypothyroidism is a condition characterized by abnormally low thyroid hormone production. There are many disorders that result in hypothyroidism or hyperthyroidism that may directly or indirectly involve the thyroid gland.

TCM suggests two patterns of Yang deficiency for hypothyroidism.

1. Spleen/kidney deficiency
2. Heart/Kidney deficiency

Pulse patterns

Deep and thin pulse or slow pulse pattern—K↓Sp↓St↓GB↓—spleen and kidney deficiency (hypothyroidism)

Deep, slippery, slow pulse pattern—H↓K↓P↓Si↓—heart and kidney deficiency (hypothyroidism)

Wiry and rapid pulse—K↓UB↑H↑Si↓ (hyperthyroidism)

Thin and rapid pulse—H↓LIV↓St↓ (hyperthyroidism)

Deep, thin, rapid pulse—K↓SP↓H↑ (hyperthyroidism)

In the treatment of thyroid problems, acupuncture can be used to restore hormonal balance, regulate energy levels, and help manage sleep, emotions, and menstrual problems. There are several powerful acupuncture points on the ear and the body that can be used to regulate the production of thyroid hormones. Treatments take all of your symptoms into account and are aimed at balancing the energy within the body to optimize health.

Objectives:

1. To provide drugless, harmless treatment.
2. To provide complete healing treatment.
3. To alleviate drug dependence.

#### **Biography** 

Sumita Satarkar was the first woman acupuncturist to represent India at the United Nations, through the All Shah Behrum Baug Society, Mumbai (recognized as having consultant status at the UN), in New York at the CSW 61 Commission on Status of Women. She was the first Indian acupuncturist to introduce the philosophy of acupuncture as a science of healing to the University of Perugia, Italy. She is the head of the School of Alternative Healing and Acupuncture Insync, Pune. Sumita Satarkar completed her PhD at the age of 39 and is completing another PhD in integrative medicine at the International Quantum University of Integrative Medicine, USA. She has presented more than 50 papers at conferences in India and overseas and has published more than 100 articles in different scientific journals. She is an international trainer in advance modalities in acupuncture. She is the author of the book *Acupuncture an Art of Healing*, which has been published in six editions and multiple languages.

### 3.21. Electroacupuncture Combined with Bone Marrow Mesenchymal Stem Cells Transplantation Inhibits Inflammation to Alleviate Glial Scar Formation in Transected Rat Spinal Cord

Ding, Y.

#### **Abstract** 

It is known that the glial scar formation resulting from spinal cord injury (SCI) impedes axonal regeneration. A glial scar contains a lot of glial fibrillary acidic proteins (GFAP) and chondroitin sulfate proteoglycans (CSPGs). Our previous study has reported that governor vessel electroacupuncture (EA) treatment combined with bone marrow mesenchymal stem cell (MSCs) transplantation downregulated GFAP and CSPG expression in a transected rat spinal cord model. However, little is known about the mechanism of EA and transplanted MSC combination downregulating GFAP and CSPGs expression. The present study investigated whether EA combined with MSC transplantation (EA + MSCs) treatment could attenuate glial scars by inhibiting the inflammatory response following SCI. The results showed that EA + MSCs treatment could facilitate M1 macrophage to M2 macrophage transition, meanwhile decreasing the mRNA and protein expression of proinflammatory factors (tumor necrosis factor-α, TNF-α and interleukin-1β, IL-1β), and increasing the mRNA and protein expression of anti-inflammatory factors (interleukin-10, IL-10) in the transected spinal cord. Moreover, EA + MSCs treatment remarkably reduced reactive astrogliosis and CSPGs accumulation after the transected spinal cord. In vitro study demonstrated that TNF-α and IL-1β could increase astrocyte proliferation and GFAP and CSPGs mRNA expression in astrocytes in a dose-dependent manner. Together, our findings indicate that EA + MSCs treatment can inhibit glial scar formation, which can be partly mediated by increasing the number of M2 macrophages to reduce the inflammatory response after SCI. EA + MSCs treatment may be a potential therapeutic strategy for the clinical treatment of SCI.

#### **Biography** 

Ying Ding, PhD, is an associate professor and Master’s supervisor for the Department of Histology and Embryology, Zhongshan School of Medicine, Sun Yat-Sen University. She is a member of the Guangdong Provincial Anatomical Society Council, the Guangdong Provincial Rehabilitation Medicine Association, and the Guangdong Provincial Experimental Medicine Professional Committee of Chinese and Western Medicine Association. Her research mainly focuses on the combined mechanism of electro-acupuncture (EA) and transplanted stem cells to repair spinal cord injuries. She found that EA could increase the neurotrophin-3 (NT-3) level and further promote the neuron-like cell differentiation, synaptogenesis, and myelin formation of transplanted bone marrow mesenchymal stem cells (MSCs) in a transected rat spinal cord model. So far, she has obtained grants from the National Natural Science Foundation, the Natural Science Foundation of Guangdong Province, and the Bureau of Traditional Chinese Medicine Science Foundation of Guangdong Province. She has published 20 SCI-E papers in total. 

### 3.22. The Mechanisms Underlying Neuropsychiatric Comorbidity—Insights from the Theory of “The Same Treatment for Different Diseases” in TCM Based on the Exploration of the Antidepressant Response of TCM/Acupuncture from Clinical Investigations and Laboratory Study

Bao, T.

#### **Abstract** 

Inflammation is considered to be a promoting pathway involved in psychiatric disorders and neurobiological abnormalities, including the imbalance of neurotransmitters, dysfunction of the immune system, disorders of the neuroendocrine system, and abnormalities of synaptic plasticity.

To give a new interpretation of the antidepressant effects of traditional Chinese medicine/acupuncture, we investigate the theory of “treatment based on syndrome differentiation” from the perspective of comorbidity and reveal the mechanisms underlying the neuropsychiatric comorbidity based on the theory of “the same treatment for different diseases” in TCM.

Clinical investigations: The effects of Chinese herbs on anxiety disorders; he effects of Chinese herbs on depressive disorders; illuminating the effectiveness and necessity of early interventions for those with depressive status and investigating the underlying mechanisms by circulating microRNA high-throughput sequencing.

Experimental investigation: A study on effective target screening for the antidepressant effects of acupuncture based on iTRAQ analysis; the mechanisms underlying the antidepressant response of acupuncture based on differentially expressed proteins in the hippocampus based on antibody protein chip technology; the mechanisms underlying the antidepressant response of acupuncture based on genome-wide transcriptome analysis by RNA sequencing.

#### **Biography** 

Tuya Bao is from Mongolia, China. She is a professor and PhD supervisor at the School of Acupuncture–Moxibustion, Beijing University of Chinese Medicine. Her main research field is clinical and mechanistic research on acupuncture in depression and stroke patients.

### 3.23. Effects and Mechanisms of Herbal Compounds on the Neurovascular Unit after Ischemia/Reperfusion

Zheng, G.-Q.

#### **Abstract** 

To date, no drug has been proven to be neuroprotective for acute ischemic stroke in clinical trials, and thus a conceptual framework—the neurovascular unit—has been proposed for investigating the pathophysiology of how brain cells die after acute ischemic stroke. Studies over the past few decades have revealed the protective and therapeutic efficacy of compound Chinese herbal medicine for acute ischemic stroke. In our group, we mainly focused on the efficacy and mechanisms of herbal compounds for neurovascular unit injury after ischemia/reperfusion, including ginseng and its active compounds, rhubarb root and rhizome and its active compounds, Astragaloside IV, borneol, and catalpol, by using preclinical systematic review and experimental research.

#### **Biography** 

Dr. Zheng is a professor of neurology and has doctorate degrees in both neurology (Western medicine) and Internal Medicine (Traditional Chinese Medicine). He is also a doubly licensed practitioner of Traditional Chinese Medicine and Western medicine. He is the vice-dean of neurology and psychiatry and director of integrative brain disease at the second affiliated hospital of Wenzhou Medical University. He has been named a young or middle-aged academic leader in Zhejiang higher education institutions, a promising young person and high-level talent in the medical community of Zhejiang Province, a young or middle-aged academic leader in Wenzhou Medical University, a key academic leader at the second affiliated hospital of Wenzhou Medical University, a reserved academic leader of medical innovation in Zhejiang Province, a 151 Talent of Zhejiang Province, and a 551 Talent of Wenzhou City. He was invited to be a peer-review expert for the Hong Kong Food and Health Bureau Fund and the National Natural Science Foundation in China. He is a committee member of 15 national and provincial associations such as the Professional Committee of Evidence-Based Medicine and Neurology in the Chinese Association of Integrative Medicine, a group leader of evidence-based neurology, the editor of seven international journals and four domestic journals, and a peer reviewer for more than 20 SCI journals such as *Annals of Internal Medicine*. He has hosted and participated in 19 fundraising events for bodies such as the NSFC, and has published 10 books and 65 SCI papers.

### 3.24. Pain Location of Primary Headache Can Be Confirmed Using Gold Acupuncture and Meridian System of Korean Hand Therapy

Park, K.H., Park, S.W., and Yoo, T.W.

#### **Abstract** 

Background: For the diagnosis of primary headache, anatomical diagnosis is needed; we mainly depend on history taking and the International Headache Classification criteria. The location of a headache can be diagnosed if we apply TCM Acupuncture and Meridian Points. The system is complicated to use. We propose a Gold Acupuncture and Meridian Point system. 

Subjects and methods: This procedure was performed during history taking, including palpation of the affected regions at the Department of Neurology, Pusan National University Hospital, from March 2009 to February 2012. Two hundred primary headache patients who have no other neurological or systemic diseases were included. We checked pain location on both sides of the head using Gold Acupuncture Points on Gallbladder Meridian (CM1-12) and Urinary Bladder (CI1-8).

Results: The headache points are grouped using the Gallbladder and Urinary Bladder Gold Acupuncture and Meridian System: the migraine headache patients belonged to Gallbladder and tension-type headache belonged to Urinary Bladder Meridian; mixed-type headaches belonged to a combined Gallbladder and Urinary Bladder Meridian. There were three pure migraine groups, three pure tension-type headache groups, and nine mixed-form headache groups.

Conclusion: The pain location of a primary headache can be easily confirmed using the Gold Acupuncture and Meridian System.

#### **Biography** 

Kyu Hyun Park, MD, PhD was a professor of neurology from 1989 to 2014 and is Emeritus Professor at Pusan National University, Busan, Korea. He works at the Department of Neurology, Jungang Nara Hospital as of 2016. He received his license to practice internal medicine in 1982 and his license of Neurology in 1984. He was a research fellow at the Clinical Electrophysiology and Muscle Laboratory UAB, USA in 1987. He is a previous president of the Korean Neurological Association. He has studied acupuncture since 1968, especially Korean Hand Acupuncture since 1982. He has membership of the National Academy of Medicine of Korea, the Korean Association of Internal Medicine, the Korean Neurological Association, the Korean Dementia Association, the International Headache Society, and the Korean Hand Acupuncture. His fields of interest are neuroelectrophysiology, cerebral blood flow, headache, stroke, movement disorder, dementia, and acupuncture, especially Koryo Hand Therapy. He has lectured on acupuncture in Beijing, Tokyo, Kyoto, Fukuoka, UCI and Spence Technology Seattle, Harvard Medical School, Graz, Austria, and The Hague, the Netherlands.

### 3.25. Applying the Computerized Diplopia Test System in Treating Ophthalmoplegia with Acupuncture

Zhou, L.

#### **Abstract** 

Ophthalmoplegia is a clinically common and refractory disease, usually secondary to brain lesions and metabolic disorders, with diplopia and eyeball movement disorder as the main symptoms. These could be catastrophic to patients’ physical and mental health and affect their quality of daily life. Recently, treating ophthalmoplegia with acupuncture, as a new kind of method with sure efficacy, has earned lots of attention from the medical community. The main purpose of this study is to introduce the application of a computerized diplopia test system in the localization of paralyzed extra-ocular muscles for adjusting the plan of acupuncture treatment. In clinical practice, selection of the area and angle of insertion as well as the electronic current intensity, monitoring the status of patients’ recovery, evaluating the curative effect, and adjusting the treatment plan could all be achieved using a computerized diplopia test system. This new system was a combination of computer technology, anatomy, and traditional acupuncture that could not only quantify the effectiveness of treatment and modernize acupuncture, but also potentially treat ophthalmoplegia.

#### **Biography** 

Professor LingYun Zhou, who has a PhD in acupuncture and moxibustion, is the chief of the Eyeball Movement Disorder Treatment Center in the first affiliated hospital of Harbin Medical University. Her research interests include clinical and fundamental investigations of treating ophthalmoplegia with electroacupuncture. Over the past 10 years, by applying innovative intra-orbital electroacupuncture therapy and computerized diplopia test system into clinical practice, more than 10,000 ophthalmoplegia patients have been cured. The team led by Professor Zhou invented a Computerized Diplopia Test System, an Eyeball Movement Measure Instrument, an electrical extra-ocular muscle stimulator, and brought in the electromyography instrument of extra-ocular muscle, making intra-orbital electroacupuncture treatment a reality and turning assessments digital. She built up the first worldwide eyeball movement disorder treatment center, which included a specialty clinic and ward. Professor Zhou has completed funded research projects through the Natural Science Foundation of China, the Foundation of the State Administration of Traditional Chinese Medicine, and provincial key funds, as well as four other projects. She holds two patents, has won six awards including the provincial science and technology progress prize and medical new technology award, and has published 32 papers, seven of them included in SCI.

## 4. Posters

### 4.1. Quantification of the Stimulation Quantity by Acupuncture Manipulation Using a Needle–Tissue Interaction Measurement System

Jung, C.

#### **Abstract** 

Background and Objectives: The stimulation amount is an important factor impacting the therapeutic effect. This study aims to quantitatively measure the difference in the amount of stimulation according to parameters of acupuncture manipulation using the acupuncture needle–tissue interaction measurement system (ANTIMS).

Methods: For the quantification of acupuncture manipulation, an ANTIMS was used in phantom tissue. The motor and force sensors of the needle insertion device were connected to the control software. To compare the difference in the amount of stimulation according to lifting–thrusting manipulation and to twisting–rotating manipulation, all needling procedures were performed by a computer controlled by an ANTIMS. The force Z exerted on a tissue was measured at various frequencies and ranges of movement by inserting a needle and the torque Z force exerted on a tissue was measured at various rotation frequencies and angles by rotating a needle with an ANTIMS.

Results: At a constant frequency of movement (0.5 Hz), Force Z according to the range of movement (2~10 mm) increased in the tissue model with increasing range of movement (*p* < 0.05). At a constant range of movement (6 mm), acupuncture needle force according to frequency of movement (0.25~1 Hz) increased with increasing frequency of movement. In the range of the rotation angle 60~180°, rotation frequency 0.05~0.20 Hz, Torque Z force of change in the tissue model is measured. At a constant frequency of the needle rotation, the rotation angle grows larger. Torque Z force was significantly increased (*p* < 0.05). At a constant angle of rotation, the frequency of the rotation angle is greater, and the Torque Z force increased significantly (*p* < 0.05).

Conclusions: Various manipulative variables for each operation in accordance with the quantitative measurement of the amount of stimulation will be required for further study. It can be based on techniques for teaching acupuncture model development and training on the proper amount of stimulation

#### **Biography** 

Chanyung Jung has been a research professor at OMD/College of Korean Medicine, Dongguk University since February 2015. She became a specialist of in March 2010 by studying in the Department of Acupuncture and Moxibustion, Dongguk University Ilsan Oriental Hospital. Her PhD in acupuncture and moxibustion, also from the College of Korean Medicine, Dongguk University, was received in February 2011. 

### 4.2. Analysis of Trends in Clinical Studies about Dizziness in the *Journal of Korean Medicine*

Yi, G.

#### **Abstract** 

Objectives: The aim of this study is to review domestic clinical studies about dizziness in Korean medicine.

Methods: Population keywords “현훈 (Hyeonhun), 현기증 (Hyeongijeung), 어지럼 (Eojireom), 어지러움 (Eojireoum), 실신 (Silsin), 眩暈 (Hyeonhun), 眩氣症 (Hyeongijeung), 失神 (Silsin)” were searched for in five database systems (DBpia, KISS, KMbase, NDSL, and RISS) from 13 September to 15 September 2016.

Sixty clinical studies were collected and classified by published journal, year, etiologic disease, study design, tools of study, study results, evaluation of disease pattern and process, interventions (herbal medicine and acupuncture treatment points), and number of studies that chose that prescription.

Conclusions: Dizziness was researched constantly since 1998 and seven clinical studies were issued in 2007, the most in one year. The journal in which the most studies were presented was the *Journal of Korean Oriental Internal Medicine* (12 studies out of 60, or 20%). Out of the 60 studies there were 12 original articles (20%) and 48 case reports (80%).

BPPV was reported as an etiologic disease in nine studies (20.45%) out of 44. VAS was used mainly as a dizziness assessment tools in studies (26 out of 38, or 68.42%). Nine studies (15.0%) out of 60 included an evaluation of the disease pattern and process.

Banhabaekchulcheonma-tang was reported as an herbal medicine prescription in nine out of 44 studies (20.45%) and ST36 was most often chosen as an acupuncture point (in 24 out of 45 studies, 53.33%). This result is not representative but does capture the present tendency in treating dizziness in Korean clinical studies.

#### **Biography** 

Gilhee Yi has a bachelor’s degree from the Department of Oriental Medicine, Dajeon University, and is now studying Oriental medicine, ophthalmology, otolaryngology, and dermatology at the College of Dongguk University, Republic of Korea.

### 4.3. A Clinical Study of Electroacupuncture and Auricular Acupuncture for Abdominal Pain Relief in Patients with Pancreatitis: A Pilot Study

Lee, Y.

#### **Abstract** 

Objectives: The purpose of this study is to evaluate the feasibility of further acupuncture research as an effective, alternative, and safe treatment for abdominal pain in patients with pancreatitis.

Methods: This study is an open-label, assessment-blind, parallel-designed pilot clinical trial. Thirty participants will be assigned to the acupuncture group (*n* = 15) or the usual care group (*n* = 15). All patients will receive the conventional standard-of-care (SOC) therapy, but only the experimental group will receive acupuncture therapy six times a week for up to 12 weeks or until the pain is resolved. For conventional SOC therapy, painkillers will be given. In the treatment group, subjects will receive the identical SOC therapy in combination with electroacupuncture therapy on 12 acupuncture points (LI4, PC6, SP6, GB39, ST36, ST37), and auricular acupuncture therapy on five auricular acupuncture points (sympathetic, Shen Men, abdomen, Pancreas gall, and spleen). The primary outcome will be measured using the visual analogue scale (VAS), and the secondary outcome will be measured using the painkiller demand, quality of life index, and severity of pancreatitis by abdominal computed tomography (CT). Assessments will be made at baseline and at weeks 1, 4, 8, and 12. Results of abdominal CT will be evaluated at baseline and at week 12.

Conclusions: The result of this trial will reveal the effectiveness and safety of acupuncture treatment for abdominal pain in patients with pancreatitis.

#### **Biography** 

Yeonsun Lee graduated from the Department of Oriental Medicine, Gachon University. She is currently completing her Master’s degree, and is also in her first year as a resident of acupuncture and moxibustion at the College of Korean Medicine, Dongguk University, Republic of Korea.

## 5. Author Affiliations

- Jing, X. Institute of Acupuncture and Moxibustion, China Academy of Chinese Medical Sciences, China
- Litscher, G. President of ISLA, Chair of the World Congress, Medical University of Graz, Austria
- Burgoon, T.S. American Academy of Medical Acupuncture, USA
- Zheng, W. University of Macau, Macau, China
- Stockert, K. the Austrian Acupunture Society, Austria
- Zhang, Z.-J. School of Chinese Medicine, The University of Hong Kong, Hong Kong, China
- Ong, E.S. Singapore University of Technology and Design, Singapore
- Suen, L.K.P. ^1^ Squina International Centre for Infection Control; ^2^ School of Nursing, The Hong Kong Polytechnic University, Hong Kong
- Bering, R. Center of Psychotraumatology, Alexianer Krefeld GmbH, Germany
- Inchauspe, A.A. National University of La Plata, Argentina
- Haid, M. Sheboygan Acupuncture LLC, USA
- Shang, C. Baylor College of Medicine, USA
- Park, K.H. Department of Neurology, Jungang Nara Hospital Busan, Korea
- Park, S.W. Department of Neurology, Bongseng Hospital, Korea
- Yoo, T.W. Korean Hand Acupuncture Therapy, Korea
- Pan, W. Shuguang Hospital Affiliated to Shanghai University of Traditional Chinese Medicine, China
- Silva-Filho, R.d.C. Brazilian school of Chinese Medicine, Brazil
- Ling, H.W. Medical Acupuncture and Pain Management Clinic, Brazil
- Xu, L. Zunyi Medical College, China
- Wu, J.-H. Ming Chun University, Taiwan
- Liu, Z. Nanhai Affiliated Maternity and Children’s Hospital of Guangzhou, Traditional Chinese Medicine University, China
- Varaei, S. Tehran University of Medical Sciences, Iran
- Guo, L. Hebei University of Technology, China
- Wang, Z.-G. Southeastern University, China
- Lü, X.-Y. Southeastern University, China
- Li, X. Chengdu University of TCM, China
- Satarkar, S. School of Alternative Healing and Acupuncture Insync (Saheacci), India
- Ding, Y. Zhongshan School of Medicine, Sun Yat-sen University, China
- Bao, T. Research Center of Mental and Neurological Disorders, School of Acupuncture-Moxibustion and Tuina, Beijing University of Chinese Medicine, China
- Zheng, G. The Second Affiliated Hospital of Wenzhou Medical University, China
- Zhou, L. The First Affiliated Hospital of Harbin Medical University, China
- Jung, C. College of Korean Medicine, Dongguk University, Republic of Korea
- Yi, G. Department of Oriental Medicine Ophthalmology, Otolaryngology and Dermatology, College of Oriental Medicine, Dongguk University, Republic of Korea
- Lee, Y. Department of Oriental Medicine Acupuncture and Moxibustion, College of Oriental Medicine, Dongguk University, Republic of Korea

